# Six-Month Outcomes from the Prospective, Multi-Center, Non-Randomized Clinical Study of the COVERA^(^™^)^ Arterio VeNous (AV) Stent Graft in the Treatment of Stenosis in the VEnous OutfloW of AV Fistula Access Circuits (AVeNEW PAS)

**DOI:** 10.1007/s00270-024-03930-7

**Published:** 2025-01-09

**Authors:** Bart Dolmatch, Talar Saber, Margo Underwood, and AVeNEW PAS trial investigators, Jonah Licht, Angelo Makris, N. Roxanne Neyra, Jeffrey Hoggard, Scott Schultz, Alexander Kurbanov, Suresh Margassery, Robert Molnar, Rajeev Narayan, Juan Carlos Perez Lozada, Reza Talaie

**Affiliations:** 1 The Palo Alto Medical Foundation, Sutter Health, Palo Alto, USA; 2 Clinical Affairs, Becton, Dickinson and Company, Tempe, USA; 3 Scientific Affairs, Becton Dickinson and Company, Tulsa, USA

**Keywords:** Arteriovenous fistula, Hemodialysis, Dialysis, Angioplasty, Stent

## Abstract

**Purpose:**

The AVeNEW Post-Approval Study (AVeNEW PAS) follows upon results from the AVeNEW IDE clinical trial and was designed to provide additional clinical evidence of safety and effectiveness using the Covera™ Vascular Covered Stent to treat arteriovenous fistula (AVF) stenoses in a real-world hemodialysis patient population.

**Materials and Methods:**

One hundred AVF patients were prospectively enrolled at 11 clinical trial sites in the USA and treated with the covered stent after angioplasty of a clinically significant target stenosis. The primary safety outcome was freedom from any adverse event that suggests the involvement of the AV access circuit evaluated at 30 days. The primary efficacy outcome was Target Lesion Primary Patency (TLPP) at six months, determined by an independent core laboratory. Secondary outcome measures included technical success defined as successful deployment to the intended location and access circuit primary patency (ACPP).

**Results:**

Safety was 94.9% with no device-related deaths nor in-patient hospitalization. Technical success was 100%. TLPP rates at 1, 3, and 6 months were 100, 89.7, and 82.2%. ACPP rates at 1, 3, and 6 months were 98, 76.3, and 60.0%. Target stenoses were 81% restenotic, and 75% located in the cephalic vein arch. There were 35% non-target stenoses treated with angioplasty during the index procedure.

**Conclusion:**

The 6-month results of the AVeNEW PAS confirm results from the AVeNEW IDE clinical trial and demonstrate safety and efficacy using the Covera^(^™^)^ Covered Stent in a real-world US hemodialysis patient population.

**Trial Registration:**

NCT04261686.

**Level of Evidence:**

3 – prospective, multicenter.

**Graphical Abstract:**

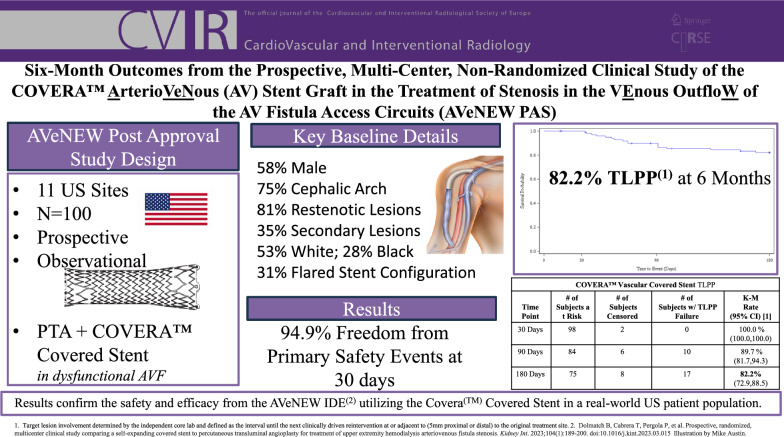

## Introduction

For over 50 years arteriovenous fistula (AVF) have been preferred for hemodialysis access. However, the development of stenosis within an AVF circuit can reduce the effectiveness of hemodialysis, lead to AVF thrombosis, and in some cases cause secondary problems including aneurysmal dilation, prolonged post-dialysis bleeding, development of pseudoaneurysms, and even delayed life-threatening bleeding [1–5].

Today, AVF stenosis is largely treated with angioplasty (PTA), but restenosis at the treatment site is frequently encountered. There are several adjuncts to PTA that have been studied with the hope of improving post-PTA durability [6–8]. We recently published results from the randomized, prospective, multi-center trial comparing the Covera™ Vascular Covered Stent (CS) to PTA for the treatment of AVF stenosis (AVeNEW IDE study) [9]. Compared to PTA alone, that study demonstrated superior target lesion primary patency at 6, 12, and 24-months when the CS was placed immediately after PTA. On March 1, 2019, the Covera™ Vascular Covered Stent was approved by the Food and Drug Administration (FDA) for treatment of stenosis in hemodialysis arteriovenous fistula (AVF) circuits with the provision that a post-approval study (PAS) would be done to further provide long-term clinical evidence demonstrating reasonable assurance of the continued safety and effectiveness with the CS. This AVeNEW PAS (NCT04261686) was undertaken on July 3, 2020 using a prospective, multi-center, US-based, non-randomized clinical study trial design, treating clinically significant stenotic lesions in the venous outflow of AVFs. We report the 6-month outcomes from this study.

## Materials and Methods

### AVeNEW PAS Study Design

One hundred patients with dysfunctional AVFs bearing a clinically significant target AVF stenosis were prospectively treated with the CS following PTA. Post-dilatation was recommended in the SG instructions for use (Table 1). The effectiveness endpoint at 6 months was TLPP and the primary safety endpoint was 30 days. Definitions are outlined in Table 2. Eleven sites in the United States (US) enrolled patients between August 2020 and October 2022.

**Table 1:** Key eligibility criteria

Key clinical inclusion criteria	Key clinical exclusion criteria
Patient must voluntarily sign the Informed Consent Form prior to collection of study data or performance of study procedures	The patient is dialyzing with an arteriovenous graft
Patient was male or nonpregnant female ≥ 21 years of age, with an expected lifespan sufficient to allow for completion of the study	The target lesion has had a corresponding thrombosis treated within 7 days prior to the index procedure
Patient had an upper extremity AVF that had undergone at least one successful dialysis session with two-needle cannulation, prior to the index procedure	The AVF is located in the lower extremity
	The patient has an infected AVF or uncontrolled systemic infection. The patient has a known uncontrolled blood coagulation/bleeding disorder
	The subject has a known allergy or hypersensitivity to contrast media which cannot be adequately pre-medicated
	The patient has a known hypersensitivity to nickel-titanium (Nitinol) or tantalum
Angiographic inclusion criteria	Angiographic exclusion Criteria
Patient must have angiographic evidence of a stenosis ≥ 50% (by visual estimation) located in the venous outflow of the AV access circuit and present with clinical or hemodynamic evidence of AV fistula dysfunction	Additional stenotic lesions (≥ 50%) in the venous outflow that are > 3 cm from the edge of the target lesion and are not successfully treated (defined as ≤ 30% residual stenosis) prior to treating the target lesion
The target lesion must be ≤ 9 cm in length. (Multiple stenoses may exist within the target lesion.)	An aneurysm or pseudoaneurysm is present within the target lesion
The reference vessel diameter of the adjacent non-stenotic vein must be between 5.0 and 9.0 mm	The location of the target lesion would require the Covera™ Vascular Covered Stent be deployed across the elbow joint
	The target lesions are located within a stent
	The location of the target lesion would require that the covered stent be deployed at or across the segment of fistula utilized for dialysis needle puncture (i.e., cannulation zone)
	The location of the target lesion would require that the covered stent be placed in the central veins or under the clavicle at the thoracic outlet
	There is incomplete expansion of an appropriately sized angioplasty balloon to its expected profile in the operator’s judgment, during primary angioplasty at the target lesion

**Table 2:** Key outcome measure definitions

Outcome measure	Definition
Safety	Freedom from any adverse event(s), localized or systemic, that reasonably suggests the involvement of the AV access circuit (not including stenosis or thrombosis) that require or result in any of the following alone or in combination: additional interventions (including surgery); in-patient hospitalization or prolongation of an existing hospitalization; or death
Target lesion primary patency (TLPP)	Interval following the index intervention until the next clinically driven reintervention at or adjacent to (approximately 5 mm proximal and distal to, by visual estimation) the original treatment site or until the extremity is abandoned for permanent access. Primary patency ends when any of the following occurs: a) clinically driven reintervention in the treatment area; b) thrombotic occlusion within the treatment area; c) surgical intervention that excludes the original treatment area from the AV access circuit d) abandonment of the AV fistula due to inability to treat the original treatment area. Vessel rupture at the target lesion caused by PTA is not a TLPP failure unless achieving hemostasis also causes thrombosis or requires treatment other than what the patient has been assigned to Clinically driven reintervention is defined as a lesion that is ≥ 50% stenosed and the presence of at least one clinical, physiological, or hemodynamic abnormality attributable to the stenosis based on the K/DOQI Guidelines. These are: decreased access blood flow (< 500 ml/min or a 25% decrease in flow), elevated venous pressures, decreased dialysis dose, prolonged bleeding, difficult puncture, infiltration, recirculation, pulling clots and/or abnormal physical exam
Access circuit primary patency (ACPP)	Interval following the index intervention until the next access thrombosis or repeated intervention. ACPP ends with a clinically driven reintervention anywhere within the access circuit; from the arterial inflow to the SVC-right atrial junction. Vessel rupture caused by PTA is not an ACPP failure unless achieving hemostasis also causes thrombosis
Acute technical success	Successful deployment, based on the operator’s opinion, of the implant to the intended location assessed at the time of the index procedure

In many ways our treatment group mirrored the experimental arm of the AVeNEW Investigational Device Exemption (IDE) trial (NCT02649946) and allowed inclusion of patients with non-target stenoses if they underwent successful PTA of the non-target stenosis before treatment of the target lesion with PTA and covered stent placement.

AVeNEW PAS was a condition-of-approval post-approval study and therefore sponsored by Bard Peripheral Vascular, subsidiary of Becton, Dickinson, and Company (BD). The study was registered on clinicaltrials.gov (NCT04261686) and the FDA Post-Approval Study Database (P170042 S002). The trial protocol was designed in conjunction with the FDA, the sponsor, and the principal investigator. Data were collected at each site by investigators using standardized web-based clinical case report forms. A clinical events committee reviewed all reported adverse events (AEs). An independent core lab reviewed and analyzed angiographic data of the AV access circuits at time of procedure and subsequent reinterventions (Yale Cardiovascular Research Group, New Haven, CT).

The study was conducted in accordance with good clinical practice standards. All procedures were in accordance with the ethical standards of the institution(s) and with the 1964 Helsinki Declaration and its later amendments or comparable ethical standards. Investigators followed a standard protocol approved by their institutional reviews boards (IRB), and all patients provided written informed consent prior to completing any procedure.

## Inclusion/Exclusion Criteria

Patients eligible for AVeNEW PAS were ≥ 21 years of age with ESKD and undergoing hemodialysis using a mature upper extremity AVF that had been cannulated successfully for hemodialysis. Enrollment required clinical or hemodynamic evidence of AVF dysfunction, and patients could be enrolled if they had recent thrombosis up until 7 days prior to the index procedure. The target stenosis had to narrow the AVF lumen diameter by 50% or more, be no longer than 9 cm, and be the culprit lesion causing AVF dysfunction. After successful PTA of any non-target stenoses, the target lesion was treated with PTA per the operator’s standard of care, confirming that the PTA balloon had reached its full expanded profile by fluoroscopic visual assessment. Vessel diameters of the non-stenosed segment had to fall between 5–9 mm, and the CS was then placed at the target lesion site with oversizing between 0.5 mm and 2 mm. It was recommended to extend the covered stent at least 5 mm into the non-stenosed segment of the vessel at both ends of the treatment site. The procedure was completed using a standard angioplasty balloon within the covered stent to assure full device expansion with final angiographic imaging of the treatment site.

Exclusions included lesions located across the elbow joint, in a cannulation zone, in a thoracic central vein, or within a previously placed stent or covered stent. The key eligibility criteria are further outlined in Table 1.

## Study Device

The Covera™ Vascular Covered Stent is a flexible, self-expanding endoprosthesis comprised of expanded polytetrafluoroethylene (ePTFE) fully encapsulating a nitinol stent framework and compatible with 8–9F sheaths. It is available in diameters 6 – 10 mm in both a straight and flared configuration. The flared configuration has an approximately 3 mm larger diameter than the body at the downstream end of the device.

## Study Outcome Measures

The clinical outcome for safety was defined as freedom from any adverse events (AEs), localized or systemic, that reasonably suggests the involvement of the AV access circuit (not including stenosis or thrombosis) that require or result in any of the following alone or in combination: additional interventions (including surgery); in-patient hospitalization or prolongation of an existing hospitalization; or death.

The performance outcome measure was Target Lesion Primary Patency (TLPP) defined as the interval following the index intervention until the next clinically driven reintervention at or adjacent to (approximately 5 mm proximal and distal to, by visual estimation from the independent core lab) the original treatment site or until the extremity is abandoned for permanent access. Key outcome measure definitions are further outlined in Table 2. All patients were followed up at 30 days, 90 days, and 6 months, with ongoing future data collection at 12 months, 18 months, 24 months, and 36 months.

## Statistical Analysis

Baseline variables and endpoints are presented with descriptive statistics. The involvement of the target lesion and access circuit patency are based upon core lab review. All other endpoints are site reported. Analyses are primarily based upon the Modified Intent to Treat (MITT) population, defined as subjects who have signed the informed consent and who are treated with the CS (following PTA). The rates are estimated using the Kaplan–Meier method. The 95% confidence intervals are estimated using Greenwood’s formula.

## Results

### Patient Demographics

Patient demographics and medical histories are further detailed in Table 3. All patients were treated in the US, and more than half of the patients were male (58%). Of the 100 patients, 53% were White and 28% Black with remaining as either Asian, American Indian, or Unknown. Twenty-five percent reported as Hispanic or Latino. Common comorbidities included hypertension (93%), diabetes (78%), dyslipidemia (59%), cigarette smoking (42%), and congestive heart failure (33%).

**Table 3:** Patient demographics and medical history

Variable		Patient (*n* = 100)	%
Gender	Male	58	58
	Female	42	42
Ethnicity	Hispanic or latino	25	25
	Not hispanic or latino	74	74
	Missing	1	1
Patients Treated	USA	100	100
Race	American indian or alaska native	5	5
	Asian	2	2
	Black or African american	28	28
	White	53	53
	Unknown	12	12
Relevant Medical Risk	Bleeding disorder	1	1
	Steal syndrome	2	2
	Aortic disease	3	3
	Valvular heart disease	3	3
	Deep vein thrombosis	5	5
	Stroke	8	8
	Transient ischemic attack	8	8
	Myocardial infarction	12	12
	Peripheral arterial/vascular disease	12	12
	Cancer	17	17
	Atrial fibrillation	20	20
	Other	24	24
	Coronary artery disease	32	32
	Congestive heart failure	33	33
	Cigarette smoking	42	42
	Dyslipidemia	59	59
	Other	62	62
	Diabetes	78	78
	Hypertension	93	93
# of Months on hemodialysis	Mean (SD)	30.7 (26.25)	n/a
	Median	22.0	
	Min–Max	1–120	

## Lesion and Circuit Characteristics

Seventy-one percent of patients had their AVF in their left arm. Ninety-seven percent did not have thrombus present at Index, but were permitted to have had previous thrombosis in their access history. Thirty-five percent of patients had secondary non-target stenoses. Eighty-one percent of the target lesions were restenotic and 55% of the target lesions had been treated within the last six months. Seventy-five percent of lesions were in the cephalic arch.

Further descriptions of the access circuit characteristics and procedural details are outlined in Table 4

**Table 4:** Access circuit characteristics and procedural details

Variable		Patient (*n* = 100)	%
Target limb	Left Arm	71	71
	Right Arm	29	29
Index procedure access site	Forearm	4	4
	Upper Arm	95	95
	Other	1	1
Thrombus present at Index?	Yes	3	3
	No	97	97
Total number of non-target lesions treated	Number of subjects	35	35
	Number of non-target lesions treated	43	n/a
Number of non-target lesions treated per subject	1	28	n/a
	2	6	n/a
	3	1	n/a
Non-target lesions treated?	# Subjects treated prior to Index	33	n/a
	# Subjects treated after Index	3*	n/a
Post-dilatation of the target lesion?	Yes	98	98
	No	2	2
Adjunctive procedures performed in the access circuit	Yes	7	7
	No	93	93
De Novo	Yes	19	19
	No	81	81
Previous interventions in last 6 months	Number of previous Interventions	109	n/a
	Number of subjects w/ Intervention	70/100	70
	Involved target lesion	55/100	55
Clinical indicator of access dysfunction	Abnormal bruit	4	4
	Decreased access blood flow	18	18
	Decreased dialysis dose (Kt/V)	1	1
	Difficult puncture	12	12
	Diminished or abnormal Thrill	27	27
	Elevated venous pressures	20	20
	Flaccid access	1	1
	Infiltration	6	6
	Prolonged bleeding	45	45
	Pulling clots	2	2
	Pulsatility	65	65
	Other	27	27
Lesion location	Basilic vein outflow	13	13
	Basilic vein swingpoint	1	1
	Cannulation zone	1	1
	Cephalic vein arch	75	75
	Cephalic vein outflow	6	6
	Juxta-anastomotic	2	2
	Other	2	2
Total number of lesions within target treatment area	Mean (SD)	1.1 (0.40)	n/a
	Median	1.0	
	Min–Max	1–3	
Reference vessel diameter (mm)	Mean (SD)	8.32 (0.906)	n/a
	Median	9.0	
	Min–Max	6.0–10.0	
Target lesion length (mm)	Mean (SD)	34.2 (22.08)	n/a
	Median	30.0	
	Min–Max	2–90	
Target lesion stenosis (%)	Mean (SD)	75.7 (11.44)	n/a
	Median	80.0	
	Min–Max	50–100	
Predilation balloon diameter (mm)	Mean (SD)	8.6 (1.00)	n/a
	Median	9.0	
	Min–Max	6–10	
Predilation balloon length (mm)	Mean (SD)	54.4 (20.9)	n/a
	Median	40.0	
	Min–Max	40–120	
Residual stenosis (%)	Mean (SD)	30.6 (22.62)	n/a
	Median	30	
	Min–Max	0–90	

^*^One subject had more than one non-target lesion and was treated before and after CS placement

The CS was predominantly used in the straight configuration (69.3%) with a diameter of 10 mm (49.5%). Stent length was up to 100 mm and nearly all lesions were post-dilated (98%). The final residual stenosis was a mean of 3.8%

## Outcomes

A total of 100 patients were treated with the CS after angioplasty. Freedom from protocol-defined primary safety events (site reported) through 30 days post-index procedure was 94.9% (93/98). The five patients who had a safety event required additional intervention: four patients had vessel spasm during the index procedure and were successfully treated with PTA and one patient had a pseudoaneurysm in the access circuit with skin thinning, requiring surgical revision. There were no device-related deaths and no in-patient hospitalizations.

Acute technical success, defined as successful deployment of the implant to the intended location at the time of the index procedure, was 100%.

**Fig. 1:**
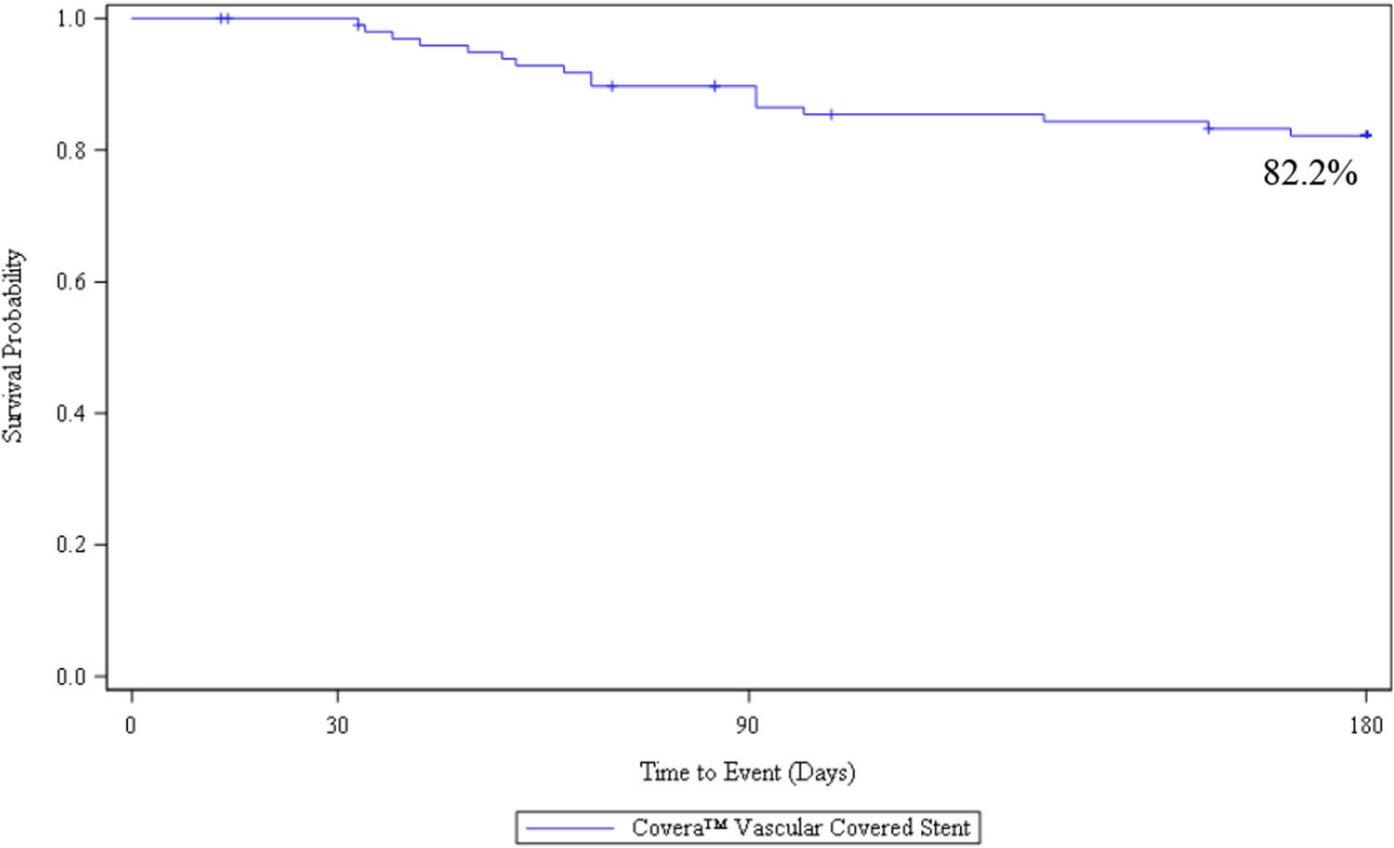
AVeNEW PAS survival analysis of target lesion primary patency (TLPP)* at 6 Months

**Fig. 2:**
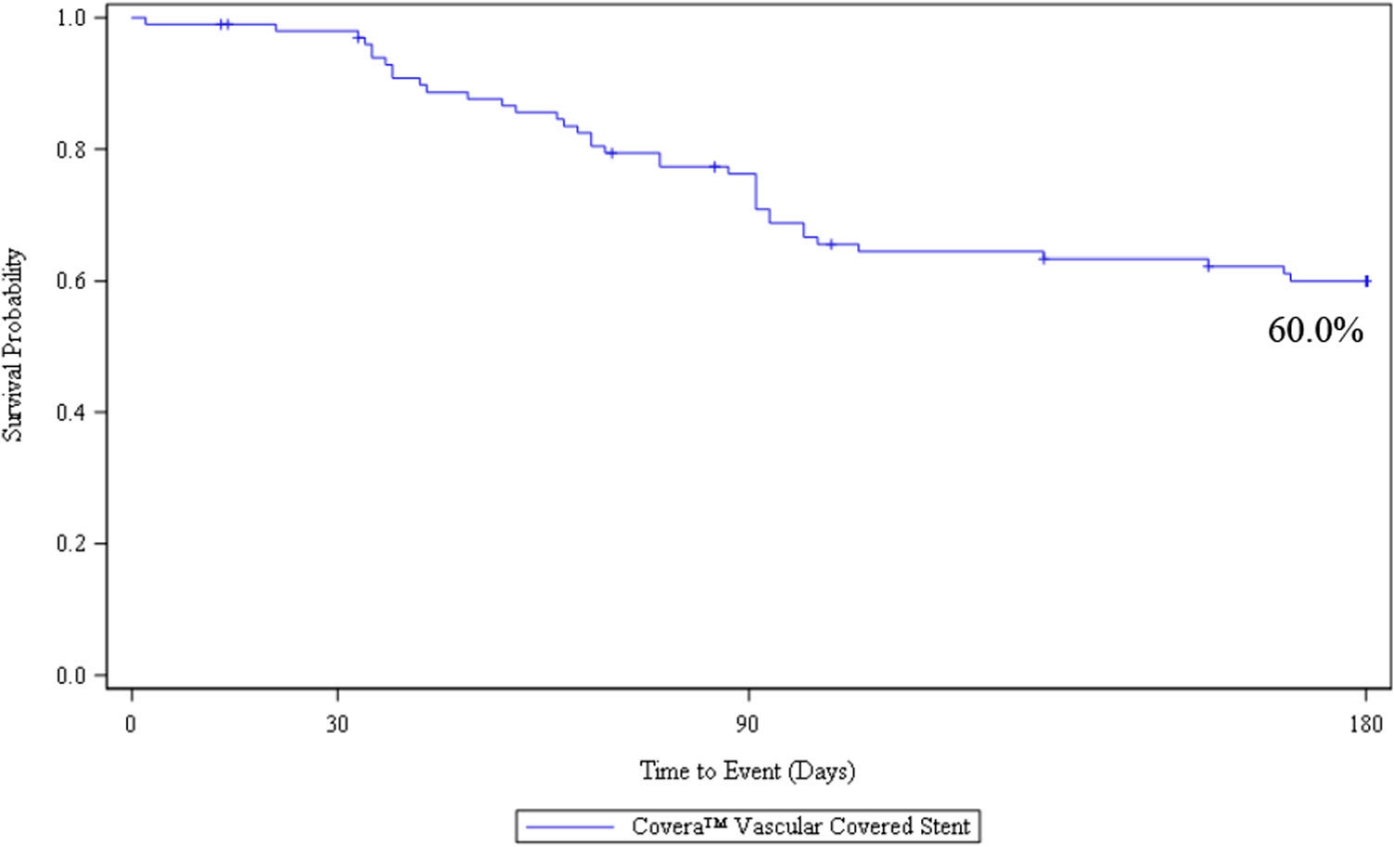
AVeNEW PAS survival analysis of subjects with Access Circuit Primary Patency (ACPP) through 6 Months

Ninety-three (93%) of patients were included in the TLPP analysis as they either completed the 6-month follow-up timepoint or had a TLPP event before they discontinued the study prior to six months. At 6 months, the Kaplan–Meier estimate of TLPP was 82.2% (Fig. 1). One of the 17 subjects lost TLPP due to access abandonment related to steal syndrome. The remaining lesions were located in the cephalic arch/cephalic outflow (*n* = 10) and in the basilic vein outflow/swingpoint (*n* = 6). All reinterventions included PTA, and three subjects were treated with a stent. At six months, 8 of the 17 subjects (47%) who had TLPP failure due to stenosis, and 21 of the 38 subjects (55%) who had ACPP failure also had a secondary lesion treated at the index procedure. At 6 months, the Kaplan–Meier estimate of ACPP was 60.0% (Fig. 2).

Of the 100 patients enrolled, 7 (7%) died within six months, and while final data collection has not been completed to one year, we know that 13 of the 100 patients (13%) were no longer alive beyond one year. The leading causes of death were cardiac disease, “ESKD,” and cessation of dialysis. No deaths were attributable to the CS.

## Discussion

The CS has been FDA approved for use in AVFs for over 5 years, conditional upon performance of a post-approval clinical study designed to assess safety and efficacy in a “real world” patient population.

The 6-month results of our post-approval study closely mirror outcomes from the AVeNEW IDE randomized clinical trial. Safety (94.9% freedom from primary safety events at 30 days) was almost identical to the results from the IDE trial (95% at 30 days). TLPP at 1, 3, and 6 months was 100%, 89.7%, and 82.2%, and paralleled results from the AVeNEW IDE CS group showing a 1, 3, and 6-month TLPP of 97.1, 90.6, and 78.7%. Simply stated, at each time point to 6-months, the results from this post-approval study in 100 patients confirmed the results from the original randomized study and support the findings of both safety and efficacy using the CS for treatment of AVF stenosis.

Drug coated balloon (DCB) studies have previously looked at TLPP in AVF with reported ranges between 68 and 97% at 6 months [6, 7, 10–16]. Coincidentally, the CS result was identical to the reported DCB rate from the In.Pact trial (82.2%) [7]. However, it would be an inaccurate statement that results were equivalent, as they have not been compared in a head-to-head trial and patient populations between these trials were disparate. As an example, the In.Pact trial had a 36.4% Asian population (vs. 2% CS), a subgroup who has reported higher patency rates post DCB [17]. This same trial also reported 17.6% of lesions in the cephalic arch vs. 75% in this CS trial, a known difficult lesion to treat [18, 19]. Definitions between the trials for TLPP were also different, further solidifying that the comparison is inappropriate.

At the time of publication, there are no other large, prospectively published clinical trials utilizing covered stents in AVF. However, interim results were presented at Cardiovascular and Interventional Radiological Society of Europe (CIRSE) on September 14, 2024 for the Merit Wrapsody AV Access Efficacy Study (WAVE- NCT04540302) with a 27% improvement of CS to PTA (89.8% vs. 62.8% *p* < 0.0001) [20]. This is similar to AVeNEW IDE results with a 30.8% improvement of CS to PTA (78.7% vs. 47.9% *p* < 0.001 [9]. There is not a comparative clinical trial between the two CS’s and endpoint definitions vary. Differences in patient populations are not fully known at the time of publication.

It is also worth noting that ACPP (primary circuit patency) was less than TLPP. At 6 months, ACPP was 60% while TLPP was 82.2%. Factors beyond target lesion patency impact circuit patency. Non-target stenoses, treated with PTA during the index procedure, were found in 35 of 100 (35%) of our patients. Approximately half of all subjects who lost TLPP or ACPP had a secondary lesion at the index procedure (47 and 55%). We believe the prevalence is due to a generous protocol definition of permitting secondary lesions greater than 3 cm from the edge of the target lesion. As demonstrated in the AVeNEW IDE randomized trial, frequent recurrence of non-target stenoses, as well as the development of new non-target stenoses, negatively impacts ACPP [9]. Optimal management of non-target stenoses remains unclear, but our findings suggest that treatment with PTA, alone, may not be the best approach given excellent TLPP results with the CS in both the AVeNEW IDE study and the AVeNEW PAS. We speculate that an approach that treats all significant stenoses with a covered stent may provide better ACPP. While this would likely increase cost at the time of intervention, it is possible that costs to the healthcare system could be economically justified due to fewer maintenance procedures, as was shown in prior studies regarding economic modeling of covered stents in AV access circuits [21, 22].

It is worth noting that three fourths of the stenoses in this PAS were in the cephalic vein arch, and 81% of all stenoses were recurrent. While other locations of stenosis, and de novo lesions, were included in this study, the results are strongly influenced by the preponderance of recurrent cephalic vein arch stenoses. Furthermore, we excluded placement of the CS across the elbow, in cannulation zones, and in the thoracic central veins. Very few were placed in forearm AVFs, and the study was designed to study the outcome in non-thrombosed AVFs. Our results represent use of the CS in a manner similar to the way it was used in the AVeNEW IDE clinical trial, and we do not make any claims regarding possible results when used in off-protocol situations.

KDOQI guidelines[4] consider it reasonable to use covered stents in AVFs for in-stent restenosis as well as for the treatment of ruptured venous stenotic segments and highly selective aneurysm/pseudoaneurysm, indications that were not considered in this study. The KDOQI guidelines did not provide guidance on the use of covered stents in dysfunctional AVF’s due to limited clinical data with small sample sizes [23, 24]. The guidelines specifically called out the need for high-quality evidence using covered stents in AVF for clinical-based outcomes. The AVeNEW IDE results, and now AVeNEW PAS, adds 242 patients with the CS to the evidence base when making decisions on dysfunctional AVF.

We report our 6-month data, but this post-approval study will continue to a final endpoint of 3 years. While long-term data are desirable, there will be a great deal of attrition due to mortality. Our interim 13% 1-year mortality rate is consistent with mortality rates from previously reported studies [9], and not unexpected given the multiple comorbidities found in ESKD patients. At two years we project that only 3/4th of enrolled patients will still be alive for analysis, many of whom will already have lost TLPP and ACPP. By 3 years, attrition and loss of patency will further diminish the number of patients for analysis. We therefore believe that our results at 6-months may be the strongest signal of safety and efficacy, reflecting the benefit using a covered stent to treat AVF stenosis in this patient population (Table 5).

**Table 5:** Study device and post dilation details

Variable	Description	Covera (*n* = 101)	Percentage (%)
Covered stent configuration	Flared	31	30.7
	Straight	70	69.3
Location of lesion treated w/ flared covered stent configuration	Basilic vein outflow	10	9.9
	Basilic vein swing point	1	1
	Cephalic vein Arch	19	18.8
	other	1	1
Location of lesion treated w/ straight covered stent configuration	Basilic vein outflow	3	3
	Cannulation zone	1	1
	Cephalic vein arch	57	56.4
	Cephalic vein outflow	6	5.9
	Juxta-anastomotic	2	2
	Other	1	1
Covered stent diameter (mm)	7	3	3
	8	29	28.7
	9	19	18.8
	10	50	49.5
Covered stent length (mm)	30	1	1
	40	25	24.8
	60	33	32.7
	80	28	27.7
	100	14	13.9
Placement configuration	Overlap	1	1
	Single	99	98
	N/A	1	1
Stenosis post-deployment (%)	Mean (SD)	8.2 (10.69)	n/a
	Median	10	n/a
	Min–Max	0.0–60.0	n/a
Placement successful at intended site	Yes	101	100%
Post-dilatation	Yes	98	
	No	2	
Post-dilatation balloon diameter (mm)	Mean (SD)	8.8 (1.00)	n/a
	Median	9.0	
	Max	7–10	
Post dilatation balloon length (mm)	Mean (SD)	54.5 (21.26)	n/a
	Median	40	
	Max	40–120	
Residual stenosis (%)	Mean (SD)	3.8 (4.82)	n/a
	Median	0	
	Max	0–10	
Post dilatation pta into healthy tissue?	Yes	89	89
	No	9	9

There are limitations to this post-approval study. By the very nature of gathering data in a real-world context, the proceduralists who participated in this study practiced without a highly prescriptive protocol. For instance, stenoses were visually assessed as being 50% or greater in diameter and associated with evidence of clinical dysfunction or hemodynamic significance. PTA was performed with balloon catheters at the operators’ best estimation of appropriate sizing, and it was recommended that the covered stent diameters should be sized within a range of 0.5-2 mm larger than the adjacent reference vessel, though measurements were not mandated. While treatment was not precise, the purpose of this study was to evaluate the CS in a real-world study where most operators do not make measurements, but rather use their clinical acumen to guide treatment decisions. However, our TLPP results were determined by an independent core lab, which we believe adds rigor to interpretation of these results.

Unlike the randomized AVeNEW IDE trial that was performed with patients from both the US and outside the US, our study only enrolled US patients. This, however, was not much of a limitation, since 92.3% of the CS patients in the IDE trial were enrolled in the US, so enrollment of patients in our study was quite similar to enrollment in the IDE randomized trial.

## Conclusion

The 6-month results of the AVeNEW PAS confirm the safety and efficacy results from the AVeNEW IDE utilizing the Covera^(^™^)^ Vascular Covered Stent in a real-world US patient population. Use of the CS for the treatment of stenotic lesions in the venous outflow of upper extremity arteriovenous fistulae provides definitive advantages for maintaining AVF target lesion patency and helps to maintain circuit patency.
