# Correction to: Six-Month Outcomes from the Prospective, Multi-Center, Non-Randomized Clinical Study of the COVERA(™) Arterio VeNous (AV) Stent Graft in the Treatment of Stenosis in the VEnous OutfloW of AV Fistula Access Circuits (AVeNEW PAS)

**DOI:** 10.1007/s00270-025-03980-5

**Published:** 2025-02-06

**Authors:** Bart Dolmatch, Talar Saber, Margo Underwood, and AVeNEW PAS trial investigators, Jonah Licht, Angelo Makris, N. Roxanne Neyra, Jeffrey Hoggard, Scott Schultz, Alexander Kurbanov, Suresh Margassery, Robert Molnar, Rajeev Narayan, Juan Carlos Perez Lozada, Reza Talaie

**Affiliations:** 1 The Palo Alto Medical Foundation, Sutter Health, Palo Alto, USA; 2 Clinical Affairs, Becton, Dickinson and Company, Tempe, USA; 3 Scientific Affairs, Becton Dickinson and Company, Tulsa, USA

**Correction to: Cardiovasc Intervent Radiol** 10.1007/s00270-024-03930-7

In the Acknowledgements, Juan Carlos Perez Lozada’s affiliation should be listed as (Yale University, New Haven, CT).

The original article has been corrected.

